# Parametric Optimization of an Air–Liquid Interface System for Flow-Through Inhalation Exposure to Nanoparticles: Assessing Dosimetry and Intracellular Uptake of CeO_2_ Nanoparticles

**DOI:** 10.3390/nano10122369

**Published:** 2020-11-28

**Authors:** Lars B. Leibrock, Harald Jungnickel, Jutta Tentschert, Aaron Katz, Blaza Toman, Elijah J. Petersen, Frank S. Bierkandt, Ajay Vikram Singh, Peter Laux, Andreas Luch

**Affiliations:** 1 German Federal Institute for Risk Assessment (BfR), Department of Chemical and Product Safety, Max-Dohrn-Strasse 8-10, 10589 Berlin, Germany; harald.jungnickel@bfr.bund.de (H.J.); jutta.tentschert@bfr.bund.de (J.T.); aaron.katz@bfr.bund.de (A.K.); frank.bierkandt@bfr.bund.de (F.S.B.); ajay-vikram.singh@bfr.bund.de (A.V.S.); peter.laux@bfr.bund.de (P.L.); andreas.luch@bfr.bund.de (A.L.); 2 Information Technology Laboratory, National Institute of Standards and Technology, 100 Bureau Drive, Gaitherburg, MD 20899-8311, USA; blaza.toman@nist.gov; 3 Materials Measurement Laboratory, National Institute of Standards and Technology, 100 Bureau Drive, Gaitherburg, MD 20899-8311, USA; elijah.petersen@nist.gov

**Keywords:** air–liquid interface system, inhalation toxicology, nanoparticles, CeO_2_, standardization, cause-and-effect analysis

## Abstract

Air–liquid interface (ALI) systems have been widely used in recent years to investigate the inhalation toxicity of many gaseous compounds, chemicals, and nanomaterials and represent an emerging and promising *in vitro* method to supplement *in vivo* studies. ALI exposure reflects the physiological conditions of the deep lung more closely to subacute *in vivo* inhalation scenarios compared to submerged exposure. The comparability of the toxicological results obtained from *in vivo* and *in vitro* inhalation data is still challenging. The robustness of ALI exposure scenarios is not yet well understood, but critical for the potential standardization of these methods. We report a cause-and-effect (C&E) analysis of a flow through ALI exposure system. The influence of five different instrumental and physiological parameters affecting cell viability and exposure parameters of a human lung cell line *in vitro* (exposure duration, relative humidity, temperature, CO_2_ concentration and flow rate) was investigated. After exposing lung epithelia cells to a CeO_2_ nanoparticle (NP) aerosol, intracellular CeO_2_ concentrations reached values similar to those found in a recent subacute rat inhalation study *in vivo*. This is the first study showing that the NP concentration reached *in vitro* using a flow through ALI system were the same as those in an *in vivo* study.

## 1. Introduction

During the last two decades, engineered nanomaterials (ENMs) have received widespread attention due to their broad range of applications including industry, [1,2,3,4,5,6,7] medicine [8,9,10] and consumer products [11,12,13]. During the manufacturing process and consumer usage of these products, there is the potential for an increased risk of inhalation exposure [14,15,16,17].

Currently, evaluation of the potential health hazards from inhalation exposure is predominantly conducted using animal models [18,19,20,21,22,23,24]. For governmental and regulatory purposes as well as based on the 3R principle (“replace, reduce, refine”), the development of alternative non-animal test methods represents a pressing issue [25]. Typically, *in vitro* nanotoxicological studies are performed under “submerged” conditions where the cells are exposed to particles dispersed in the overlying cell culture medium containing a mixture of proteins and other biological compounds [23,25,26]. However, dispersing nanoparticles (NPs) in cell culture media might alter their physical and chemical properties such as their agglomeration status and the adsorption of serum proteins onto the particles, which subsequently might impact the resulting toxicological data [27,28]. In contrast, for aerosol exposure using air–liquid interface (ALI) systems the NPs can be dispersed in water. Only after aerosolization and deposition on cells will the NPs come in contact with the cell microenvironment such as mucus or epithelia lining fluid [29].

Overall, ALI approaches can mimic *in vivo* inhalation experiments of airborne nanomaterials more closely than *in vitro* studies using submerged conditions [29]. In recent years, different ALI systems have been developed for nanomaterial testing *in vitro* [30,31,32,33,34]. The Vitrocell exposure system is one of the most commonly used commercially available ALI exposure systems for inhalation toxicity testing [29]. It has been successfully used to examine the effects of cigarette smoke [35,36,37], NPs [38,39,40,41], and diesel exhaust [30,42,43]. However, despite the increasing use of ALI systems, there is no standard operation procedure (SOP) on how NP exposure should be performed to ensure robust and reproducible results among different laboratories [29,30,39,40,42,44]. Furthermore, there are few comprehensive studies on the technical challenges of such systems (e.g., deposition efficiency, exposure time), and how to identify or overcome them. In the current literature, generally only a minimal description of the exact setup (e.g., relative humidity, temperature of the aerosol flow, or the distance between air inlet and cells) is given and a description of experimental challenges encountered is rarely provided [37,40,41,42,44,45]. This hampers the comparison among *in vitro* results achieved with such systems and with *in vivo* data [46,47,48].

A primary aim of this study was to evaluate parameters expected to influence cell viability during cell exposure using an ALI flow through system. Therefore, the human alveolar epithelia cell line A549, which was derived from a human adenocarcinoma in 1973 [49], was exposed to clean filtered air, and five parameters (relative humidity, flow rate, aerosol temperature, exposure duration, and CO_2_ supply) were varied to evaluate the impact of these parameters on the cell viability from the air exposure itself. Although A549 cells are not a primary cell line, their use here is appropriate as the main focus is to assess the robustness of the ALI system itself [41]. Using conditions that avoided a decrease in cell viability, cells were then exposed to CeO_2_ NPs. The deposition and intracellular particle uptake were characterized using inductively coupled plasma-mass spectrometry (ICP-MS) and time of flight-secondary ion mass spectrometry (ToF-SIMS) and then compared with previously obtained *in vivo* data.

## 2. Materials and Methods

### 2.1. Cell Culture

A549 cells (obtained from ATCC; catalog number: CCL-185) were cultured in Dulbecco’s Modified Eagle Medium (DMEM) supplemented with 10% fetal bovine serum (FBS) (PAN-Biotech GmbH, Aidenbach, Germany), 1% penicillin/streptomycin (PAN-Biotech GmbH, Aidenbach, Germany) and 1% L-glutamine (PAN-Biotech GmbH, Aidenbach, Germany). Cells were passaged two times per week. *In vitro* experiments were conducted with the passages 19–79. Mycoplasma contamination was tested regularly and was always negative; details about this method are described in Supplementary Materials Tables S1 and S2.

### 2.2. Cause-And-Effect Analysis

A cause-and-effect (C&E) analysis was performed on a cell viability assay for NP exposure using the ALI flow through exposure system to reveal the expected key sources of variability in the protocol. C&E analysis is a conceptual process that can help guide robustness testing and determine process control measurements that should be included in a protocol to support control charting of important sources of variability. This approach has been recently used to support the development and evaluation of several nanotoxicity assays: a cell viability assay using a submerged exposure system with A549 cells [50,51], a suite of four *in vitro* nanobioassays that measure endpoints that can be impacted in cells through oxidative stress [52], and the use of an International Standardization Organization (ISO) *Caenorhabditis elegans* assay with ENMs [53,54].

### 2.3. Characterization of the Vitrocell Exposure System (12/3 CF Module): Determination of Relative Humidity and Temperature Inside the Exposure Chamber

To assess the relative humidity in the exposure gas (clean filtered air or aerosol) an ALMEMO 2590-2A system with a FHAD 46-C0 sensor (Ahlborn GmbH, Holzkirchen, Germany) was used. The sensor was directly placed into the aerosol flow between the exposure chamber and the aerosol delivery system above the chamber by cutting a small hole in the tubing and sealing the sensor inside the tube. To understand the environmental conditions for the cells inside the exposure chamber, a second sensor (type K thermocouple PeakTech^®^ TF-56, PeakTech Prüf- und Messtechnik GmbH, Ahrensburg, Germany) was used to assess the aerosol flow temperature inside the exposure chamber using a multimeter (digital multimeter, DM01M, TACKLIFE). The sensor was placed into the tube as described above for the relative humidity measurements and then moved further into the exposure chamber. Due to its small size, the sensor could be placed slightly above the insert membrane inside the exposure chamber. To avoid a bias in the measurements from contact with medium, the sensor was not placed in direct contact with the transwell membrane which overlays the medium.

### 2.4. Parameter Optimization to Improve Cell Viability and Exposure Time

**Figure 1:**
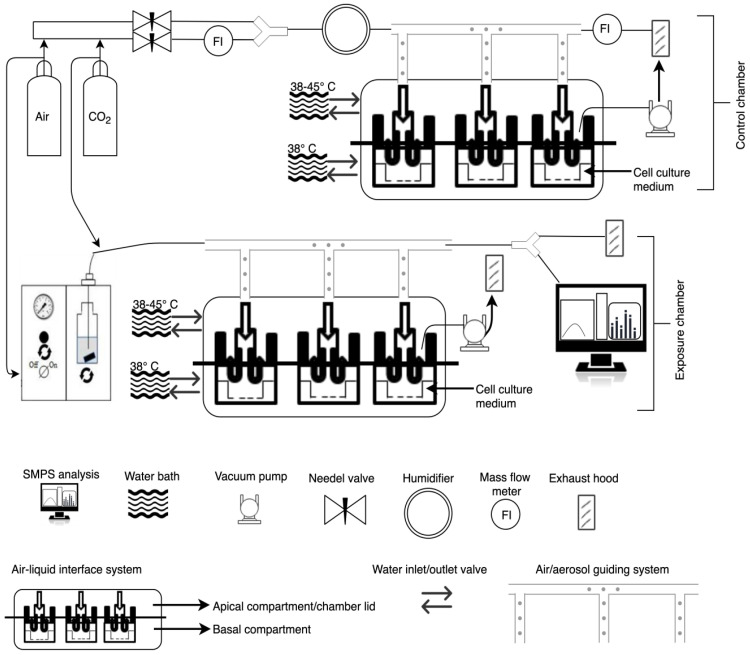
Schematic diagram of the different components of the air-liquid interface setup, aerosol mixtures, and temperature flow units.

Five different parameters (exposure duration, relative humidity, temperature, CO_2_ concentration, and flow rate) were identified by the C&E analysis as factors that may impact cell viability during ALI exposure in the flow through system and experimentally evaluated. To isolate the impact on the parameter adjusted, the cells were only exposed against clean filtered air (exposure system air negative control). The following protocol settings were used unless otherwise stated: a water bath connected to the exposure chamber and the chamber lid with a temperature of 38 °C, an exposure duration of 1 h, a total flow rate within the aerosol guiding system of 150 mL/min, and a flow rate of 5 mL/min on each insert. The air flow was guided through a glass pipe featuring three outlets on the bottom where the exposure chambers were connected (Figure 1). The flow rate was controlled by a mass flow meter (Aalborg, Orangeburg, New York, NY, USA) at the end of the pipe. A vacuum pump was used to generate the insert flow and the individual insert flow rates were adjusted using needle valves and a mass flow meter (Aalborg, Orangeburg, New York, NY, USA). The distance between the cellular monolayer and the air inlet was measured and set to 4 mm based on a spacer delivered by Vitrocell. To maintain a stable pH in the absence of 5% CO_2_, the cell culture medium (using the same composition as described before for cell culture) was supplemented with 2% (*v*/*v*) Hepes (PAN- Biotech GmbH, Aidenbach, Germany). Cell viability was compared to the incubator control for which the cells were added to the inserts at the same time and with the same cell concentration as those exposed in the ALI system.

The relative humidity was monitored and ranged from below 10% up to ≥90% depending on the setup. The relative humidity was adjusted by guiding the air through a humidifier (Gasmet Technologies GmbH, Karlsruhe, Germany) before introducing it into the glass pipe and the exposure chamber. The lid temperature was set to room temperature.

When the effect of the temperature of the exposure chamber lid was analyzed, the temperatures tested were room temperature (21 °C), 38 °C and 45 °C, while the relative humidity was set to <10%. To control the lid temperature, a second water bath was connected to the lid and set at different temperatures. As the exposure chamber is not include in a headed box, room temperature on the outer side of the chamber and tubes resulted in a condense moisture in the tubes guiding the air from the chamber exit to the exhaust. As the chamber is not transparent, condensation conditions inside the chamber (at the air inlet exit) cannot be evaluated.

To assess the impact of the flow rate on cell viability, different flow rates (1 mL/min, 5 mL/min, or 10 mL/min) were investigated. For these experiments, the relative humidity was set to >90% and the lid temperature was set to 38 °C.

The impact of the CO_2_ concentration was tested by supplementing the clean air with 5% CO_2_. Adding CO_2_ to the clean air flow was conducted in front of the humidifier and was monitored by a mass flow meter (Aalborg, Orangeburg, New York, NY, USA) (7.5 mL/min CO_2_ and ≈140 mL/min air). To ensure a 5% CO_2_ supply, the final flow rate was monitored at the exhaust of the glass pipe with a second mass flow meter (Aalborg, Orangeburg, New York, NY, USA). Needle valves were used to adjust the flow rates for each gas. For investigating the impact of adding CO_2_, the relative humidity was set to >90%, the lid temperature was set to 38 °C, and a flow rate of 5 mL/min was used.

After optimizing the aforementioned parameters, the exposure time was varied between 1 h, 2 h, 4 h and 8 h to determine the maximal exposure time during which no significant loss in cell viability was induced by clean air exposure. ALI setup conditions were set to relative humidity >90%, 5 mL/min flow rate, 38 °C lid temperature and 5% CO_2_. Overall, as described in more detail in the discussion section, an exposure of A549 cells with a flow rate of 5 mL/min, a lid temperature of 38 °C, a relative humidity of >90% and 5% CO_2_ supply were found to represent the optimal exposure conditions among those tested in this study.

In addition to the negative controls (incubator control, clean air control), a positive control was tested to confirm the dynamic range of the WST-1 (water soluble tetrazolium-1) assay. The basal compartment was filled with the culture medium spiked with Triton -X 100. Afterwards the cells were placed inside the exposure chamber and exposed to a water-based aerosol (without NPs, relative humidity >90%, 5 mL/min flow rate, 38 °C lid temperature and 5 % CO_2_).

### 2.5. Cell Viability

To evaluate the viability of the A549 cells using the exposure conditions described in the preceding section, a WST-1 assay was conducted to assess the metabolic activity of the cells. The principle of this assay is based on the stable tetrazolium salt WST-1 which is cleaved into a soluble formazan dye by cellular mechanisms including NAD(P)H-dependent oxidoreductases and dehydrogenases. Thus, the amount of formazan dye formed, directly correlates with the number of metabolically active cells in the culture [55]. A549 cell number per 12 well insert (cat. number 353180, Corning B.V., Amsterdam, Netherland; 0.4 µm pore size, 1.12 cm^2^ diameter) was 50 000. After ALI exposure of cells to air or an ENM aerosol, cells were rinsed once with 500 µL phosphate buffered saline solution (PBS) (PAN-Biotech GmbH, Aidenbach, Germany), and 300 µL fresh cell culture medium (without phenol red) containing 10 % (*v*/*v*) WST-1 reagent (4-[3-(4-iodophenyl)-2-(4-nitrophenyl)-2H-5-tetrazolio]-1,3-benzene disulfonate) (Roche Diagnostics GmbH, Mannheim, Germany) was added. After 30 min incubation at 37 °C, three technical replicates (each 50 µL) of the supernatant per insert were transferred into individual wells in a new 96 well plate and the absorbance was measured with a Tecan (GENios) plate reader (Tecan Deutschland GmbH, Crailsheim, Germany) at a wavelength of 450 nm. The absorption was also measured at a second wavelength of 562 nm, a wavelength outside the spectrum of the WST-1 probe, to evaluate for potential interferences such as bubbles. Cell viability was compared to the incubator negative control using the following equation:

(1) $$Percentage\;viability=\frac{\left(test\;parameter\;-\;medium\;blank\;control\right)}{\left(negative\;control\;-\;medium\;blank\;control\right)}\;\times \;100\%$$

where test parameter is the absorbance value of the parameter tested. Medium blank control represents the absorbance value of the test media (reagents but without cells), and the negative control represents the absorbance value for the cells in the incubator and not exposed to the ALI system.

### 2.6. Nanoparticle Dispersion and Characterization

NM-212 NPs were purchased from Joint Research Center (JRC) (JRC, Ispra, Italy). The Ce content in this material is about 81.6% [56] and the oxidative state of the CeO ion is 93.1% and 6.9% for CeO^4+^ and CeO^3+^, respectively [56]. Further details about the composition of NM-212 can be found in the JRC report [56]. The particle dispersion was prepared in accordance with the protocol of the NANOGENOTOX SOP with slight modification [57]. In summary, the following protocol was used. NM-212 were weighed and the particles were prewetted in 50 µL of 99% ethanol before being dispersed in MilliQ water to a final stock concentration of 2.5 mg/mL (10 mL final volume). Subsequently, the particle dispersion was sonicated for 5 min and 9 s (Sonoplus HD 220/UW 2200, Bandelin, Germany) to avoid particle aggregation as described in the NANOGENOTOX dispersion protocol [57]. This sonication duration allowed for a specified amount of power to be applied NM containing dispersion. For all experiments, particle dispersions were freshly prepared. Particle characterization (transmission electron microscopy, nanoparticle tracking analysis, dynamic light scattering, zeta potential and selected area electron diffraction (SAED)) of particle dispersions prepared with this method were conducted. The analytical methodology was described in detail previously [58,59]. SAED data were obtained by a JEM-2100HR transmission electron microscopy (JEOL, Tokio, Japan).

### 2.7. Particle Exposure to A549 Cells

A customized VITROCELL 12/3 CF module (Vitrocell GmbH, Waldkirch, Germany) was used to expose A549 cells to CeO_2_ NPs (NM-212) (Figure 1). A Palas VAGF 2.0 aerosol generator (Palas GmbH, Karlsruhe, Germany) operated at 1 bar inlet pressure was used to produce the particle aerosol. Cells were exposed for 1 h, 2 h or 4 h. Exposure was performed under the following conditions: a flow rate of 5 mL/min. Basal and apical compartments of the exposure chamber were heated to 38 °C. The relative humidity was >90%. The inlet distance to the cells was 4 mm. The nominal particle concentration of the aerosolized samples was 250 µg/mL. The medium volume under the insert was 3.175 mL.

### 2.8. Aerosol Characterization

A scanning mobility particle sizer (SMPS, TSI Model 3083, CPC Model No. 3775, TSI Incorporated, Shoreview, MN, USA) was used to analyze the particle size distribution and the particle mass concentration in the aerosol. Due to their electrical mobility, particles are divided in different fractions which then can be counted. Based on this information (particle number and size fraction), the particle size distribution is determined. The particle mass can be calculated based on the particle size distribution and particle number concentration.

The instrument was running with an aerosol flow to sheath flow ratio of 1/10. Operating mode was set to “low” which uses an aerosol flow of 0.3 L/min. CeO_2_ NP density was assumed to be 7.3 g/cm^3^ [18]. The inlet pressure of the aerosol generator in terms of the CeO_2_ aerosol particle size distribution was examined by adjusting the inlet pressure directly on the generator (Figure 1).

### 2.9. Determination of Intracellular Uptake of NM-212

For the determination of the particle deposition as well as the intracellular particle uptake, inductively coupled plasma mass spectrometry (ICP-MS) was used. 50 000 A549 cells were seeded per 12 well insert (cat. number 353180, Corning B.V., Amsterdam, Netherland; 0.4 µm pore size, 1.12 cm^2^ diameter). After 48 h, the basolateral medium was changed, cells were washed once with PBS, transferred onto ALI conditions, and cultured for 24 h. Subsequently, cells were used for the ALI exposure experiments.

A549 cells were exposed to a CeO_2_ NP aerosol for 1 h, 2 h or 4 h, and placed back in the incubator for 24 h (post exposure time). Subsequently, cells were washed two times with PBS (each 0.5 mL). The wash solution as well as the basolateral medium was collected and subsequently microwave digested as described in the following section.

### 2.10. Microwave Digestion and ICP-MS Analysis

Microwave digestion was conducted as described previously [60]. In brief, the membranes from the cells exposed to CeO_2_ NPs and the incubator negative control were separated from insert using a scalpel and transferred into a digestion tube. 2 mL MilliQ water, 2.5 mL HNO_3_ (69% *v*/*v*) (VWR, Darmstadt, Germany) and 1 mL H_2_O_2_ (30% *v*/*v*) (Merk, Darmstadt, Germany) were added to this tube. For the washing solution and the basal medium, 1 mL MilliQ water was added to 1 mL wash solution or 1 mL basal medium, respectively, before adding HNO_3_ and H_2_O_2_. The collected samples were digested in a microwave (MLS ultraCLAVE 2; MLS GmbH, Leutkirch, Germany).

To analyze the Ce amount using ICP-MS, we used an in house validated method based on a Ce reference material (BCR 667) [60]. ^140^Ce was quantified using a respective Ce calibration based on an ionic Ce standard solution (VWR International LTD, Leicestershire, England). ^103^Rh was used as internal standard. The recovery of this method was within the range of 86% to 120% [61]. The limit of detection (LOD) and limit of quantification (LOQ) were calculated as 3 and 10 times the standard deviation of the blank samples, respectively. Background levels of Ce were determined by blank measurements (see Table S3 for details). All cerium samples were blank-corrected by subtracting the average value of six blank samples from the measured sample concentration.

ICP-MS measurements were conducted with an iCaP-Q (ThermoFisher GmbH, Dreieich, Germany) or a Thermo Scientific XSERIES II, (Thermo Fisher Scientific, Waltham, MA, USA). For ICP-MS calibrations, LOQ’s and LOD’s are shown in Figure S6 and Table S3, respectively. Particle deposition rates were calculated by adding the ICP-MS concentration measured intracellularly and the concentrations in the washing solution and the basal medium.

### 2.11. Calculation of Deposition Efficiency

The deposition efficiency was calculated from the SMPS data and ICP-MS results. Here, the obtained particle aerosol concentration was converted to mass per surface area and the maximum deposition was determined by using Equation (2).

(2) $$Maximum\;Deposition=\frac{aerosol\;concentration\;\times \;flow\;rate\;\times \;exposure\;time}{insert\;surface\;area}$$

The measured deposition was determined using the following Equation:

(3) $$Measured\;Deposition=\frac{Deposited\;CeO_{2}\;concentration}{insert\;surface\;area}$$

The deposition efficiency was calculated by Equation (4).

(4) $$Deposition\;efficiency=\frac{Measured\;Deposition}{Maximum\;Deposition}\;\times \;100\%$$

### 2.12. Time of Flight-Secondary Ion Mass Spectrometry (ToF-SIMS)

To confirm the intracellular uptake of CeO_2_ NPs into A549 cells, ToF-SIMS measurements were carried out. Cells were exposed to the CeO_2_ NPs containing aerosol for 1 h or 4 h under optimized exposure conditions as described in the section above. After exposure, the cells were placed back in the incubator for 24 h. Subsequently the cells were rinsed twice with 0.5 mL PBS and fast frozen in liquid propane using a cryoplunger device (EMS-002, Electron Microscopy Sciences, Hatfield, PA, USA).

ToF-SIMS depth profiles were acquired using a ToF-SIMS V instrument (ION-TOF GmbH, Münster, Germany) with a 30 keV nano-bismuth primary cluster ion beam source (Bi)_x_^(y+)-^ with a Bi_Mn_ emitter [62]. The ion currents were 0.5 pA at 5 kHz using a Faraday cup. A pulse of 0.7 ns from the bunching system resulted in a mass resolution that usually exceeded 9000 (full width at half-maximum) at m/z < 500 in positive ion mode. The primary ion dose was controlled below 10^12^ ions × cm^−2^ to ensure static SIMS conditions. Charge compensation on the sample was obtained by a pulsed electron flood gun with 20 eV. The primary ion gun scanned a field of view of 200 μm by 200 μm applying a 512 pixel by 512 pixel measurement raster. Once the primary ion gun was aligned, a ToF-SIMS mass spectrum was generated by summing the detected secondary ion intensities and plotting them against the mass channels. The analytical methodology was described in detail elsewhere [63,64,65,66,67]. All depth profiles were performed in dual beam mode on the ToF-SIMS V instrument of the reflectron-type, equipped with a 30 keV bismuth liquid metal ion gun (LMIG) as primary ion source, a 20 keV argon gas cluster ion source both mounted at 45° with respect to the sample surface and an electron flood gun. Bi^3+^ was selected as primary ion by appropriate mass filter settings. Primary and sputter ion currents were directly determined at 200 μs cycle time (i.e., a repetition rate of 5.0 kHz) using a Faraday cup located on a grounded sample holder. The scanning area for analysis was 200 µm by 200 μm with 512 by 512 pixels. The sputter area for each measurement was 1000 µm by 1000 µm. Surface charging was compensated by flooding with low energy electrons. ToF-SIMS depth profiles were acquired in positive ion mode. The mass scale was internally calibrated using a number of well-defined and easily assignable secondary ions (C_2_H_5_^+^, C_3_H_7_^+^ and C_4_H_9_^+^) keeping the error in calibration for all spectra below 5 µg/mL. The data were evaluated using the Surface Lab software (ION-TOF GmbH, Münster, Germany).

### 2.13. Statistical Analysis

Statistical calculations were performed using a Markov Chain Monte Carlo Bayesian analysis to evaluate if the percentage viability was less than 100% and if the different treatment conditions were statistically equal (the null hypotheses). A Bayesian model [68,69] was applied using Markov Chain Monte Carlo programmed in OpenBUGS [70]. All measurements were assumed to be Gaussian. We used the usual Gaussian prior distributions for all the means, and Half Cauchy distributions for all the unknown variances [69]. We calculated the percentage viability with 95% uncertainty bounds for each treatment condition and plate and for the consensus values among the three plates; an example of the R code used is provided in the Supporting Information. Data was not available for the solvent system for one of the three plates for two conditions. Given the relatively small variability among the plates for the solvent system values compared to that for negative control and test condition values, the solvent system data for another plate for the same tested conditions was used for the statistical analyses for these two plates. If the MCMC of some of the posterior distributions did not converge when evaluating the consensus values, as occurred for one condition, the NIST consensus builder program (https://consensus.nist.gov/app/nicob) was used instead using the mean and standard uncertainty values calculated for each plate.

Statistical analysis of the ToF-SIMS data was performed as described in detail elsewhere [63,64,65,66,67]. In brief, the acquired data were binned to 1 mass unit (u). Data processing was carried out with the statistical package SPSS + (version 21) (IBM Deutschland GmbH, Ehningen, Germany) using the mass range between 200 mass units and 1200 mass units to detect significant differences between treated and untreated cells. Ions lower than 200 mass units were excluded from the study to avoid contaminating ions from salts, system contaminants, and other medium components; ions from the CeO_2_ particles are much larger than this range and would not be expected to impact these results. Each acquired spectrum was then normalized, setting the peak sum to 100%. A Principal Component Analysis (PCA) was performed using all ions. To show that data sets could be separated with a supervised model from each other, a Fisher’s discriminant analysis was performed (*n* = 6) (Figure S5). The performance of the discriminant model was verified by applying the cross-validation procedure based on the “leave-one-out” cross-validation formalism. * = *p* > 0.05 was considered as significant.

## 3. Results

### 3.1. Cause-And-Effect Analysis

**Figure 2:**
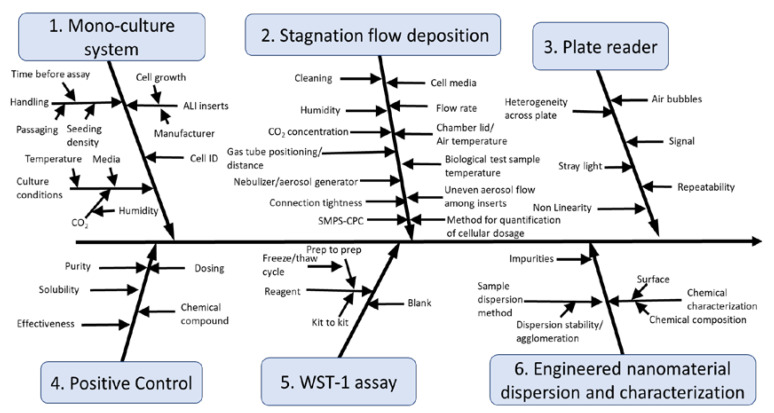
Cause-and-effect (C&E) diagram for WST-1 assay.

The C&E analysis revealed six main branches (Figure 2): cell maintenance and seeding, instrument performance, plate reader, positive control, WST-1 assay, and engineered nanomaterial dispersion and handling. These sources of variability were similar for branch 1 (cell maintenance and seeding), branch 3 (plate reader), branch 4 (positive control), branch 5 (WST-1 assay) and branch 6 (engineered nanomaterial dispersion and handling) to previous C&E diagrams prepared for the 3-(4,5-dimethylthiazol-2-yl)-5-(3-carboxymethoxyphenyl)-2-(4-sulfophenyl)-2H-tetrazolium (MTS) nanocytotoxicity assay with A549 cells [50,51,52]. Important sources of variability in branch 1 (cell maintenance and seeding) revealed during analysis of the MTS nanocytotoxicity assay were the cell number and the cell identity as some of the culture were composed of cells missing an allele and had a different toxicity to the positive chemical control [50]. Branch 3 relates to the performance of the plate reader, and therefore factors that determine the plate reader performance such as its calibration and evaluating the homogeneity across the test plate to avoid systematic biases are critical. The positive chemical control (branch 4) can serve multiple functions such as evaluating the assay sensitivity and its dynamic range. Thus, it is important to choose a positive chemical control that fulfills the measurement assurance functions for the positive chemical control for each particular assay. The important factors for branch 5 (WST-1) relate to the performance of the WST-1 assay reagents similar to those for the MTS assay. These sources of variability can be evaluated using two in-process control measurements: the blank control (medium + WST-1 assay reagents only) and the absorbance data for the incubator negative control, and can be plotted using control charts to monitor their performance across time. One key source of variability in branch 6 (engineered nanomaterial dispersion and characterization) is the dispersion procedure and NP characterization. It is known that reproducible sample dispersion methods are critical as is thorough characterization using orthogonal methods of the dispersion to confirm that the dispersion has the expected characteristics [51]. The sole branch for the WST-1 assay using exposure with the ALI system that is substantially different from those for the MTS assay was branch 2 (exposure system). Compared to the simpler exposure approach, namely pipetting, in the MTS assay which tested nanomaterial toxicity using submerged culture conditions, branch 2 is substantially more complex for the flow through ALI exposure system. A different set of process control measurements and robustness evaluation are needed for this system. This difference is the main reason why the robustness testing performed in this manuscript focused predominately on the sources of variability in branch 2. An overview of the different in-process control measurements used and evaluated in this assay is provided in Table 1.

### 3.2. CeO_2_ NP Dispersion Characterization

Given the importance of characterization of the nanomaterial dispersion (branch 6), the hydrodynamic diameter of the CeO_2_ dispersion was analyzed using two different methods, nanoparticle tracking analysis and dynamic light scattering, which revealed a hydrodynamic diameter of 180 nm ± 8.1 nm and 220 nm ± 16.6 nm after three measurements of the same suspension (values are mean ± standard deviation), respectively. The dispersed CeO_2_ NPs had a positive surface charge of 13 mV ± 1.1 mV. Further details of the nanomaterial characterization such as TEM analysis can be found in Figure S1 or here [58].

### 3.3. Evaluation of the Impact of Different Parameters in the ALI Exposure System on Cell Viability

**Figure 3:**
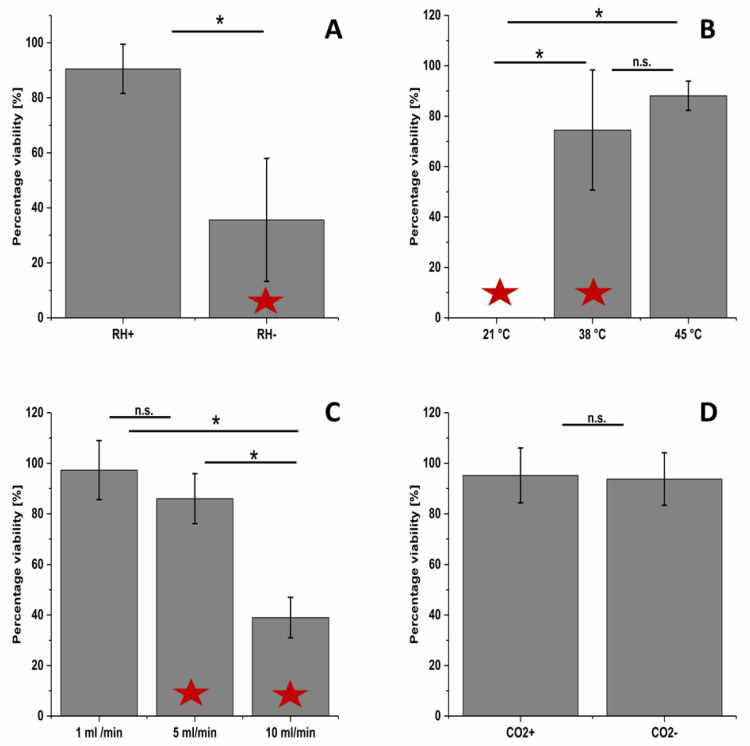
Parametric optimization of the Vitrocell exposure 12/3 CF module. Parameters affecting cell viability after 1 h clean air exposure: Relative humidity (**A**), lid temperature (**B**), flow rates (**C**) and 5% CO_2_ supply (**D**). Percentage viability values are the consensus values calculated for all three plates using the Bayesian modeling. The values are the means, and the error bars the standard deviations. n.s. = not significant. * = *p* > 0.05. Red asterisks indicate that the consensus value is significantly less than the incubator control with a 95% likelihood using the Bayesian modeling.

To better understand the factors that influence the cell viability using this exposure system (branch 2), five parameters were evaluated (Figure 3). Changing the relative humidity from <10% to >90% resulted in a significant increase in cell viability from 35% up to 90% (Figure 3A); data for the relative humidity under various exposure conditions are shown in Figure S3A. Heating the lid to 38 °C resulted in a cell viability of 75% compared to about 1% at room temperature (Figure 3B). A further temperature increase in the lid temperature up to 45 °C showed no significant difference in cell viability compared to 38 °C (corresponding air flow temperature data are shown in Figure S3B); however, the data at 45 °C among the three different plates was more consistently close to 100% viability than that for 38 °C (Figure S7B). An increase in the flow rate from 1 mL/min to 5 mL/min or 10 mL/min led to a decrease in cell viability from 97% to 86% or 39%, respectively (Figure 3C). It was decided to set the flow rate to 5 mL/min in the optimal exposure conditions, because a higher flow rate is expected to yield a higher deposition concentration even though there was a statistically significant decrease in the percentage viability compared to the incubator negative control. To emulate the *in vivo* conditions even closer and mimic the gas conditions in the alveoli, 5% CO_2_ was added to the exposure gas, which showed no decrease in the cell viability compared to the incubator negative control during a 1 h exposure duration (Figure 3D). However, for longer exposure periods, it is expected that the buffering effects of CO_2_ would have a greater impact.

**Figure 4:**
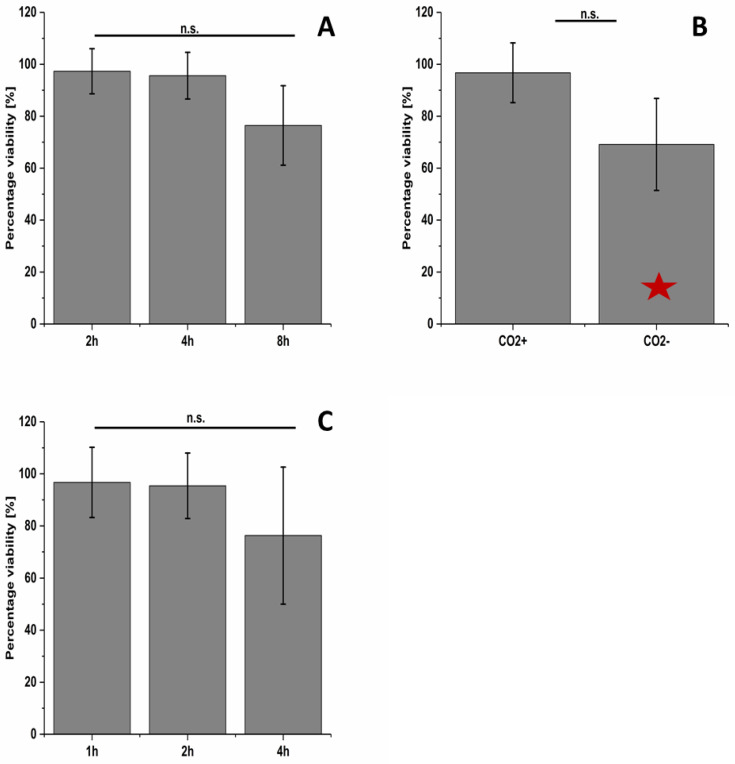
(**A**) Time-dependent cell viability for MilliQ water aerosol exposure (without ENM) using the optimized Vitrocell setup. (**B**) shows the effect of 5% CO_2_ supply to the air on the cell viability after 4 h MilliQ water aerosol exposure using the optimized Vitrocell exposure setup. (**C**) exhibits a time-dependent NM-212 exposure compared to MilliQ water aerosol (without NP) exposed cells. Percentage viability values are the consensus values calculated for all three plates using the Bayesian modeling. The values are the means and the error bars the standard deviations. n.s. = not significant. Red asterisks indicate that the consensus value is significantly less than the incubator control with a 95% likelihood using the Bayesian modeling.

An exposure of A549 cells with a flow rate of 5 mL/min, a lid temperature of 38 °C, a relative humidity of >90% and 5% CO_2_ supply were found to represent the optimal exposure conditions among those tested in this study. Using these optimized parameters, the impact of exposure time up to 8 h on cell viability was investigated (Figure 4A). The results show stable cell viability over a period of up to 4 h. For exposure for 8 h, there was no statistically significant drop in viability (76% viability) among the treatment conditions for the consensus value, but two of the three plates did have values significantly less than the incubator negative control. Exposing the A549 cells to aerosolized MilliQ water for 4 h (ALI exposure system negative control) caused a significant decrease compared to the incubator negative control for the exposure without CO_2_ but was not observed when CO_2_ gas was added (Figure 4B). During these analyses, multiple experimental challenges were encountered that required troubleshooting of the exposure system to resolve. These pitfalls and suggested solutions are listed in the Table S4. In addition, results are provided for each of the three plates to show the day-to-day variability when performing the assay (Figures S7 and S8).

Afterwards, lung epithelia cells (A549) were exposed to CeO_2_ NPs (NM-212) using the optimal exposure conditions identified in this study. No decrease in cell viability was detected after NM-212 exposure for 1, 2 or 4 h for the consensus values (Figure 4C), but a significant decrease was observed for one of the plates for the 4 h exposure (Figure S8C).

Two incubator in-process control measurements were consistently measured to evaluate the assay performance: the incubator negative control (branches 1, 3, and 5) and blank control (branch 5). An exposure system positive chemical control was performed using 0.2% Triton X-100 to evaluate the dynamic range of the assay. A decrease in cell viability down to about 6% after exposure for 1 h to 1% after 2 and 4 h exposure was observed. This data was not significantly different than the medium blank indicating a complete loss of cell viability and that the full dynamic range of the assay could be consistently achieved.

**Figure 5:**
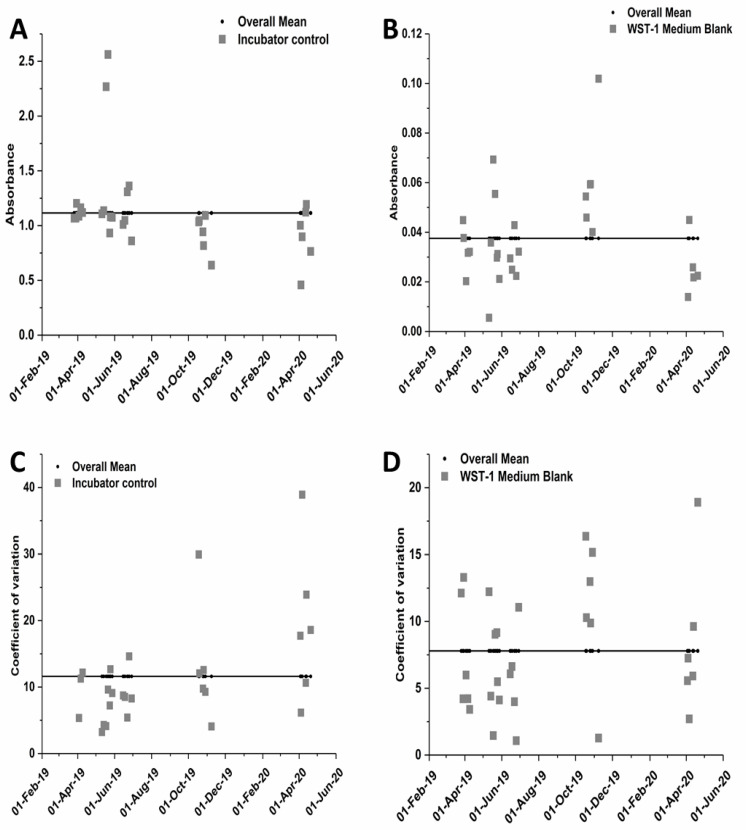
Control charting data for the WST-1 assay for the negative incubator control (**A**) and the medium blank control (**B**). (**C**,**D**) represents the coefficient of variation for all experiments depending on the date they were performed for the incubator control and the medium blank control, respectively.

Control charts were made to investigate the consistency of the WST-1 assay performance for the incubator negative control and the blank control (Figure 5). The mean absorbance value for the medium blank control was approximately 3% of that of the incubator negative control. Plotting the coefficient of variation (COV) values shows the range of variabilities among individual experiments. The mean value for the COV for the incubator negative control cells was approximately 12% (Figure 5C), while the mean value for the COV for the medium blank control was 7.5% (Figure 5D), suggesting that pipetting cells is more variable than pipetting just the medium.

### 3.4. Characterization of the CeO_2_ NP Aerosol

**Figure 6:**
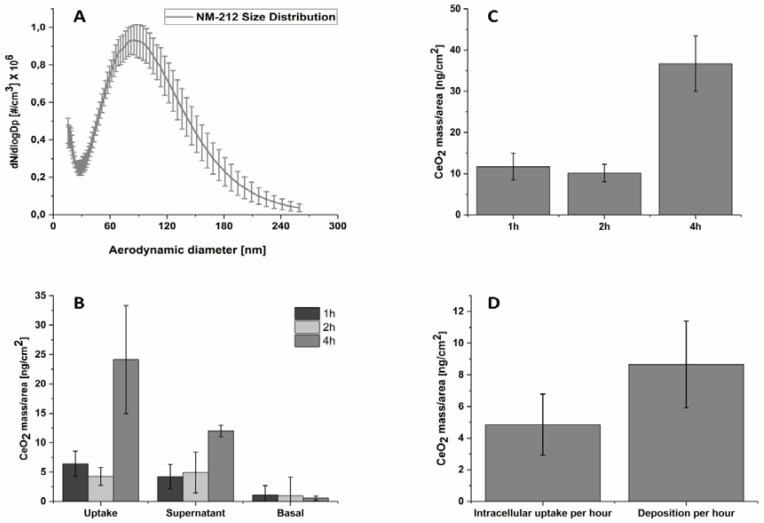
Inductively coupled plasma mass spectrometry (ICP-MS) analyses: The particle size distribution of NM-212 at 1 bar (*n* = 9). Three independent experiments with 3 data points each. Data are combined and shown as mean ± SD in (**A**). NM-212 particle uptake (**B**) and deposition (**C**) of A549 cells after air-liquid interface exposure at different time points revealed a time dependent behavior. (**D**) displays the mean intracellular uptake and mean deposition for A549 cells per h × cm^2^ after air-liquid interface exposure. (*n* = 9 = three independent experiments with 3 wells for each data point).

One of the key factors for branch 2 (exposure system) is characterization of the aerosolized CeO_2_ dispersion with different instrument settings. For example, the effect of the inlet pressure of the aerosol generator on the particle size distribution of a 250 µg/mL CeO_2_ NP solution was examined. From 1.0 bar to 1.5 bar inlet pressure, an increase in particle number was observed. However, no further increase in particle number was detected at a higher inlet of 1.8 bar (Figure S4). To avoid cell damage by static pressure into the ALI chamber, 1 bar was used for all cell exposure experiments (pressure was measured by a flow meter which is part of aerosol generator). Furthermore, the particle size distribution of NM-212 at 1 bar showed a bimodal pattern with a first maximum at approximately 20 nm and a second maximum at about 90 nm (Figure 6A). Measuring only MilliQ water showed a peak at about 25 nm that extended to up to approximately 50 nm. Additionally, a strong variability of the MilliQ size distribution was observed when measuring MilliQ solutions on different days. Therefore, a background subtraction was challenging and data under 50 nm were excluded.

### 3.5. Intracellular Uptake and Localization of CeO_2_

After characterization of the aerosol, the particle deposition and the intracellular particle uptake were analyzed by ICP-MS after exposing A549 cells to a CeO_2_ NP containing aerosol (generated using a 250 µg/mL dispersion and a pressure in the aerosol generator of 1 bar) for 1 h, 2 h or 4 h. There was an increase in particle deposition and uptake over time (Figure 6B,C). A mean intracellular CeO_2_ content of 4.85 ng × cm^−2^ × h^−1^ ± 1.93 ng × cm^−2^ × h^−1^ and a CeO_2_ deposition of 8.66 ± 2.74 ng/cm^2^ × h^−1^ were detected (Figure 6D). Furthermore, about 50% of the deposited particles were found to be intracellular. Given that dead cells might also internalize particles, the cells were washed twice with PBS to remove dead cells and particles that are weakly membrane bound.

To determine the complete particle deposition, this washing solution was also analyzed, and these results are shown in Figure 6B for the supernatant (washing solution + surfactant). The overall particle deposition includes particle deposition from dead and living cells whereas the uptake shows only the deposition and internalization by living cells. SMPS data revealed a mean aerosol concentration of 1.07 ± 0.34 mg/m^3^ for the whole size distribution and 1.03 ± 0.35 mg/m^3^ for the size distribution containing only particles bigger than 50 nm. A 100% particle deposition (5 mL/min flow rate) would therefore correspond to a theoretical, maximum aerosol deposition rate of approximately 0.29 µg/cm^2^ × h^−1^ for the whole size distribution or 0.28 µg/cm^2^ × h^−1^ for the size distribution of particles only bigger than 50 nm. Using Equation (4), a deposition efficiency of 2.98% for the whole size distribution or 3.09% for the size distribution of particles only bigger than 50 nm was achieved.

**Figure 7:**
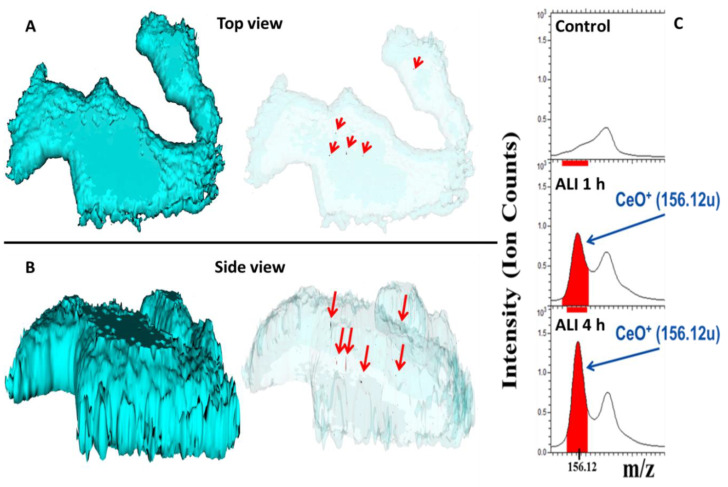
Reconstructed 3D ion images of A549 cells indicate CeO_2_ agglomerates after 4 h of air-liquid interface exposure within the tissue section (200 µm by 200 µm). The blue cell outline represents the cell membrane based on the C_3_H_8_N^+^ signal. (**A**) shows the top view while (**B**) represents the side view. (**C**) shows ToF-SIMS mass spectra (positive mode) of A549 cells exposed for 1 h or 4 h in the air-liquid interface system, showing the CeO^+^ peak in red color at m/z 156.12u. The upper spectrum shows unexposed control cells (clean air exposure). The x-axis shows the molecular weight; the y-axis the ion intensities for the peaks (*n* = 6).

ToF-SIMS 3D depth profiles for 1 h and 4 h exposure were generated to evaluate cellular uptake using an orthogonal method. Both time points show a strong CeO^+^ peak in the corresponding acquired mass spectra indicating the presence of CeO_2_ NPs within the cells (Figure 7C). Moreover, a significant increase of particle uptake from 1 h exposure (ca. 0.9 × 10^3^ ion counts) to 4 h (ca. 1.4 × 10^3^ ion counts) was observed. The reconstructed 3D ion images of the ToF-SIMS data reveal CeO_2_ agglomerates within the tissue section (Figure 7A,B, red arrows). No membrane associated CeO_2_ agglomerates could be found, thus suggesting intracellular localization of the CeO_2_ particle agglomerates in A549 cells after ALI exposure.

**Figure 8:**
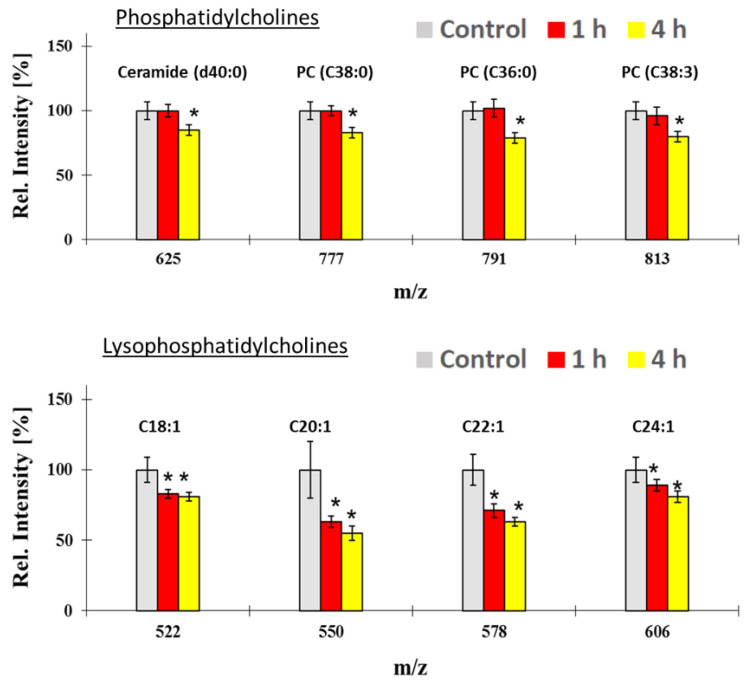
TOF-SIMS analysis of cell membranes composition changes of A549 cells after air-liquid interface exposure to CeO_2_ NPs for 1 h and 4 h. The histograms show comparisons of ion yields for characteristic cell membrane lipids for the different exposure times. For the relative intensity, the mean of the control group (clean air) of unexposed A549 cells was taken as 100% in all cases. *: *p* ≤ 0.05. Data represent the mean and error bars the standard deviation values (*n* = 6).

In addition to NP uptake, distribution and metabolic effects, we assessed molecular alterations of the cell membrane constituents by ToF-SIMS analysis that have been caused during ALI exposure of A549 cells to CeO_2_ NPs. Unexposed A549 cells were used as controls (clean filtered air ALI exposure). The results indicate a significant reduction of the lipid phosphatidylcholine biosynthesis. Down regulation of the lysophosphatidylcholine series C18:1, C20:1, C22:1 and C24:1 (Figure 8, bottom) has been detected already after 1 h of exposure. By contrast, significant downregulation of the biosynthesis of phosphatidylethanolamine and its precursor, i.e., phosphatidylcholine, was only visible in A549 cells exposed for 4 h (Figure 8, top). All the following ion assignments were done tentatively, since certified reference materials were not available. Ion m/z 791 was attributed to phosphatidylcholine PC (C36:0), ion m/z 813 to phosphatidylcholine PC (C38:3). Ion m/z 777 was matched to the applicable library spectrum of phosphatidylethanolamine PE (C38:0). Additionally, the downregulation of the ceramide biosynthesis (ion m/z 625) after 4 h of exposure could be correlated to ceramide (d40:0).

## 4. Discussion

This study provides a conceptual evaluation of key sources of variability that were identified using C&E analysis (Figure 2) of a cell viability assay with an ALI flow through exposure system. This analysis then guided the in-process control measurements used in the protocol (Table 1) and the robustness testing performed. Our first experiments with a Vitrocell 12/3 CF module and A549 cells revealed a cell viability of about 45% after 1 h of clean air exposure (Figure S2B). Such a high decrease in viability in the air negative control system would severely limit the ability of this exposure system to evaluate potential toxicological impacts from aerosol exposure to substances such as ENMs. By systematically evaluating five potential sources of variability in the ALI exposure system (branch 2), experimental settings were determined which enabled longer exposure duration for the A549 cells (more than 4 h) while maintaining >85% cell viability when compared to the incubator negative control. Our results suggest that the relative humidity, lid temperature, flow rate, CO_2_ concentration and exposure duration are crucial parameters for an ALI flow through exposure system given their impact on the cell viability (Figure 3 and Figure 4). Controlling only the cell medium temperature below the cells was not sufficient to create an adequate environment for the cells. Instead, the lid temperature also had to be controlled. These findings are in accordance to a previous study that also found that the relative humidity and aerosol flow temperature impacted the exposure system negative control cells [71]. Given the impact that these factors were shown to have an impact on the exposure system negative control cells, it is strongly recommended for other researchers to report these parameters in the methods section of papers to enable comparability of results.

Overall, nine in-process control measurements were tested to carefully evaluate key sources of variability in the assay each time it was performed to increase confidence in the measurement results (Table 1). Several of these in-process control measurements were performed in the exposure chamber while others were performed in the incubator. The positive control, which tested cells using 0.2% Triton X-100 spiked to the medium and exposed to a MilliQ aerosol, was evaluated to confirm the dynamic range of the assay. One consideration for future development of the positive control would be to test a broader range of Triton X-100 concentrations to yield a dose-response curve that could be used to ensure the consistency of the assay sensitivity, or to identify a positive control that can be nebulized and then exposed to cells using the aerosol generated. To further optimize this control measurement, a highly toxic ENM like ZnO NPs [72,73] could be used and exposed to the cells under ALI conditions to generate an “positive control”. Incubator negative control cells and the medium blank controls were also evaluated to assess the reproducibility of the WST-1 assay and of the cell pipetting on the inserts.

Using the optimal exposure setup, an exposure time up to 8 h could be achieved without a statistically significant decrease in cell viability for the consensus values (Figure 4A). Nevertheless, a trend to lower cell viability can be seen when exposing the cells longer than 4 h. Moreover, an evaluation of all three plates individually for the 8 h exposure condition revealed two of the three to be statistically less than the incubator negative control (Figure S8A). In future experiments, a 6 h time point should be evaluated to better assess the maximum exposure duration without a decrease in the viability for an individual plate. When examining the consensus values for the NM-212, there was a trend for a decrease in cell viability for the longest exposure time (4 h) even though the consensus value did not have a statistically significant decrease. There were differing results among the three plates (Figure S8C) and testing a greater number of plates would be needed to clarify if there is a real trend. Repeating experiments more than once provides more robust data, which is an important aspect in method validation [40,41,63,71,74].

To evaluate assay performance and day-to-day variability of the process control measurements, control charts were plotted (Figure S7). The mean value for COV of the incubator negative control (12%) is similar to the median average deviation values, which were typically less than 10%, obtained in a previous interlaboratory study with the MTS assay using submerged exposure conditions and A549 cells [50]. This suggests that the variability from pipetting cells is similar regardless of whether they are cultured in submerged conditions or on an ALI insert. This type of analysis helps to reveal the consistency of the data over time and should be considered when developing assay specifications that indicate the assay is preforming as expected. Combining the results from assays performed on different days, for this study from three different days, helps to account for day-to-day variability and leads to more robust statistical evaluations. Additional statistical analysis can help reveal the extent to which increasing the number of incubator negative control wells or blank control wells would improve the assay’s precision.

The particle size distribution measured by SMPS showed a bimodal shape for the CeO_2_ aerosol. When evaluating the aerosolized MilliQ water, only the first peak was detectable which is probably from residuals like salts [75,76,77]. However, NM-212 NPs have a broad size distribution ranging from below 10 nm to more than 100 nm [56]. Thus, it might be that NM-212 NPs size distribution partly contributes to the first peak. Moreover, a strong variability in the residual peak in terms of particle number concentration was seen during different experiments which caused a high background. Therefore, two calculations were performed to avoid a possible particle mass underestimation. First, the residual peak was not subtracted from the whole size distribution when determining the deposited dose. For a second more conservative approach, particles measured for the aerosolized CeO_2_ NP dispersion below 50 nm were not included when calculating the amount of deposition, because it was not possible to distinguish between the particle concentration contribution from the NPs and that from the MilliQ water. There was only a reduction of about 3% in total aerosol mass using the second approach compared to the first approach; this result is understandable because the smallest particles only had a minimal contribution to the total mass. In this particular setup of the ALI system, online measuring of the particle size distribution during the exposure experiments was not possible due to different tubing sizes of the ALI system and the SMPS system. To characterize the aerosol, the tubing was adjusted by guiding the aerosol through a glass tube. For cell experiments, the original steel tubing system from Vitrocell was used to ensure optimal performance (avoiding particle interactions with the glass wall). Another option is to measure the particle size distribution before and after the exposure. This could reveal if there was a change in the exposure concentration or particle size concentration during the course of the experiment, which may occur since NPs are known to agglomerate. Nevertheless, the deposition efficiency in this study was approximately 3%, which is in good agreement with the literature where the deposition efficiency for similar ALI systems has been reported to be between 1% to 2% [30,74,78,79].

To assess the toxicity of ENMs, quantification of the intracellular concentration is needed to facilitate the comparison between different *in vivo* or *in vitro* ENM exposure scenarios. An *in vivo* rat inhalation exposure study using CeO_2_ NPs (NM-212) revealed that the particles were exclusively found to be intracellular located [80]. This is in agreement with our ToF-SIMS data where CeO_2_ NPs were detected exclusively intracellular in A549 cells after ALI exposure and were not membrane associated (Figure 7). Thus, the washing protocol was sufficient to remove NPs which were solely present on top of the cells, or alternatively, the cells fully internalized the NPs prior to the washing procedure. ICP-MS analysis showed that of the deposited dose only half of the NPs could actually be found in the exposed A549 cells. The other half of the NPs were either located on the cell surface or in cells, such as dead cells, that were removed by the washing procedure. Exposing the human alveolar cell line A549 to a CeO_2_ NP containing aerosol (1.07 ± 0.34 mg/m^3^) an intracellular uptake rate of 4.85 ng × cm^−2^ × h^−1^ ± 1.93 ng × cm^−2^ × h^−1^ was achieved. This is similar to the subacute whole body rat *in vivo* inhalation study from Keller et al. (2014) [22] where 4.76 ng/cm^−2^ × h^−1^ was found (authors reported a lung deposition of 2620 µg NM-212/rat lung for a dose of 25 mg/m^3^; assuming a mean alveolar surface area of 50 d to 100 d old rats with 4584 cm^2^ [81] and an exposure time of 120 h) [22]. Due to their higher deposition rates compared to flow through systems, cloud chamber systems can be considered for experiments where higher deposited masses of NPs are needed than can be obtained using flow through systems [31,82]. Furthermore, more sophisticated 3D cell models amenable to longer exposure periods than systems with a single adherent cell type can help to investigate long-term effects via repeated exposure under low dose and high dose conditions [37,83].

The potential toxicological effects of NP exposure were tested using the WST-1 assay and also a ToF-SIMS metabolic interactions assay. A reduction in cell viability was not found after CeO_2_ treatment in WST-1 assay. As this assay represents an overall mean value of the metabolic activity of a cell population, its sensitivity to detect minimal adverse effects coming from low dose exposure might be limited. Thus, TOF-SIMS investigations were conducted to enhance the sensitivity of metabolic analysis to detect possible adverse effects on a molecular level [84]. With this, a reduction in phospholipids composition within the bilayer was found (Figure 8). Since the CeO_2_ NPs tested are positively charged, it is possible that washing of particles which could lead to a loss of lipids because of lipid attachment onto the particles, but this will likely be a minor effect since the fraction of the cell surface area that is covered by particles (approximately 8 × 10^−4^) is much smaller than the change in the lipid composition. In addition, other recent studies of human macrophages exposed to silver NPs have also reported changes in the phospholipid pattern [67]. Both studies show that different lipids can be affected differently after ENM exposure similar to what was observed in this study. At the moment, the exact mechanism(s) how NPs affect the cell membrane lipid bilayer composition is yet not fully understood. Therefore, more studies are needed to fully provide mechanistic explanations of the lipid changes in general as well as for single lipids. Moreover, as the A549 cell line resembles a carcinogenic phenotype, the toxicological response might be different compared to primary cells [41]. Therefore, further studies should also consider using primary cells to more closely mimic the *in vivo* environment.

To the best of our knowledge, this is the first report describing the use of an ALI system with a flow through exposure system to generate an intracellular NP concentration comparable to those observed *in vivo*. This is an important step in the development of complementary methods for inhalation studies using flow through systems. A valuable next step for future work would be to use this assay to further establish physiological relevance by evaluating a broader range of nanomaterials, especially those known to cause toxicity at lower concentrations, and compare those results to *in vivo* data for the same materials. The performance with this assay could also be evaluated with different adherent cell lines or 3D cell constructs.

## 5. Conclusions

ALI systems are considered to be a promising exposure system to study toxicological effects of airborne nanomaterials instead of *in vivo* inhalation studies and have been widely used to assess the toxicology of nanomaterials in recent years. However, the robustness of these methods is not yet well understood. Here we reported a C&E analysis of a commonly used flow through ALI exposure system. This led to a systematic evaluation of key parameters of a frequently used ALI system that could influence cell viability results and the incorporation of nine in-process control measurements into the measurement protocol. Furthermore, this ALI case study provides a robust setup to standardized ALI approaches which can be useful for regulatory context where standardized and validated *in vitro* methods are needed. Furthermore, we showed that this ALI system is able to deposit concentrations to human lung epithelia cells that result in an intracellular NP uptake similar in quantity to uptake rates observed in an *in vivo* rat study [22]. These results support the potential standardization of ALI-based exposure methods. The general procedure reported here may help to improve the standardization of the ALI *in vitro* exposure approach better enabling comparability between experiments.

## Figures and Tables

**Table 1:** Different in-process control measurements for the WST-1 ALI exposure assay.

Control Measurement	C&E Diagram Branch(es)	Procedure	Purpose
Medium blank control	Branches 3, 5	Measure the signal in wells without cells but with the WST-1 reagents	Evaluate the plate reader performance, & signals for the WST-1 reagents
Cell dosage	Branch 2	Quantify the test substance (in this case, CeO_2_ NPs) associated with the cells to evaluate the deposited dose and the intracellular concentration	Evaluate the amount of the test substance that comes into contact with the cells and is internalized; evaluate the homogeneity in the dosage among inserts
Positive control	Branches 1, 4, 5	Expose cells to air flow only (no aerosolized chemicals or particles) in the ALI system after adding a 0.2% *v*/*v* concentration of Triton-X 100 to the basal medium	Evaluate the dynamic range of the assay
Exposure system negative control	Branches 1, 2	Expose cells in the exposure system to air flow only (no aerosolized chemicals or particles) and then evaluated with the WST-1 assay	Evaluate the potential for a decrease in viability compared to the air flow only
Incubator negative control	Branches 1, 5	Evaluate the number of cells in wells not exposed to chemicals and kept in the incubator	Evaluate if a consistent number of cells have been added to the inserts, evaluate the performance of the WST-1 reagent
Relative humidity	Branch 2	A humidity sensor was used to monitor the gas prior to reaching the cells	To evaluate the impact of humidity on the exposure system negative control
SMPS-CPC	Branches 2, 6	Analyze the aerosol generated using SMPS and CPC	Characterize the NP size distribution, number concentration and mass concentration in the produced aerosol
Temperature sensor (air)	Branch 2	Use a thermocouple to measure the air temperature prior to reaching the cells	Evaluate the impact of air temperature on cell viability for the exposure system negative control
Temperature sensor (lid)	Branch 2	Use a thermocouple to measure the temperature on the insert where the cells are located	Evaluate the impact of the temperature on the insert on the cell viability for the exposure system negative control
Interference control reading	Branch 3	Measure the signal in wells at a second wavelength (562 nm) which is outside of the absorption spectrum of the WST-1 reagent	Evaluate each well for potential interferences (e.g., bubbles)

## Supplementary Materials

The following are available online at https://www.mdpi.com/2079-4991/10/12/2369/s1. Figure S1: Nanoparticle size characterization. (A) TEM image shows the size and shape of CeO2 NPs. (B) Selected area electron diffraction (SAED) of CeO2 NPs. (C) Dynamic light scattering (DLS) analysis of the particle diameter calculated via size distribution by numbers. (*n* = 3 = three independent experiments, each experiment was performed in triplicates). Out of three, two of them were overlapping and the 3rd one covered the purple one. Figure S2: A549 cell viability. (A) shows cell viability under ALI and submerged (LL) culture conditions (no ALI exposure) compared to ALI cultured cells. (B) exhibits the cell viability after 1 h ALI clean air exposure compared to the incubator control without any optimization of the ALI system (start conditions). Percentage viability values are the consensus values calculated for each of the three plates using the Bayesian modeling. The values are the means and the error bars the standard deviation. Figure S3: Characterization of the Vitrocell exposure 12/3 CF module. The relative humidity of the exposure air with and without humidification of the clean air, or a water droplet aerosol (MilliQ aerosol), which was used for particle exposure, is shown in (A). Heating the lid temperature to a certain degree does not necessarily mean that the aerosol flow temperature is equal to the lid temperature. Therefore, the relationship between the temperature of the air flow and the water bath temperature, which was used to heat the lid, is shown in part (B) for the particle exposure module and the control module (clean air only). *n* = 3 independent experiments with 1 technical replicate each. Figure S4: The particle size distribution of NM-212 as a function of the inlet pressure of the aerosol generator (*n* = 9 = three independent experiments with 3 data points each. Data from each experiment are combined and shown as mean ± SD). Figure S5: Fisher’s linear discriminant of ToF-SIMS analysis (*n* = 6). Different groups show distinct differences. Figure S6: ICP-MS calibration curves of the three different measurements. The solid lines indicate a linear regression fit to the data. Figure S7: Plate to plate analysis of consensus values for the different parameters affecting cell viability: Relative humidity (A), lid temperature (B), flow rates (C) and 5% CO_2_ supply (D). Percentage viability values are the consensus values calculated for each of the three plates using the Bayesian modeling. The values are the means and the error bars the standard deviation. Black asterisks indicate that the consensus value is significantly less than the incubator control with a 95% likelihood using the Bayesian modeling. Figure S8: Plate to plate analysis of consensus values for the different parameters affecting cell viability: (A) Time-dependent cell viability for MilliQ water aerosol exposure (without ENM) using the optimized Vitrocell setup. (B) Effect of 5% CO_2_ supply to the air on the cell viability after 4 h MilliQ water aerosol exposure using the optimized Vitrocell exposure setup. (C) A time-dependent NM-212 exposure compared to MilliQ water aerosol (without NP) exposed cells. Percentage viability values are the consensus values calculated for each of the three plates using the Bayesian modeling. The values are the means and the error bars the standard deviation. Black asterisks indicate that the consensus value is significantly less than the incubator control with a 95% likelihood using the Bayesian modeling. Table S1: Mycoplasma test, PCR compounds. Table S2: Mycoplasma test, PCR conditions and protocol. Table S3: ICP-MS LOD/LOQ levels of the three different measurements. Table S4: Possible pitfalls during an ALI exposure experiment.
