# Supplementary material for: Parametric Optimization of an Air–Liquid Interface System for Flow-Through Inhalation Exposure to Nanoparticles: Assessing Dosimetry and Intracellular Uptake of CeO2 Nanoparticles
Source: Nanomaterials (Basel). 2020 Nov 28;10(12):2369. doi: 10.3390/nano10122369 (PMC7760223; doi:10.3390/nano10122369)
Supplement: Supplementary file 1 [file nanomaterials-10-02369-s001.pdf]

nanomaterials

Supplemental Information

Supplemental Methods

R-Code:

#The code requires that both R and OpenBUGS be installed, both are free to download.
#R can be obtained at http://www.R-project.org/ ,
#OpenBUGS at http://www.openbugs.net/w/Downloads.
#The only input to the program that is needed are the values in linedata.NP is the number of plates, NI is the number of inserts.
#Pasting the entire program below into an R window will produce the estimates of the statistics.
If you run it with the debug =T in the bugs statement you can see if the MCMC has converged by looking at the trace plots.

```
##########################################################################################
library(R2OpenBUGS)

############## type data here:
linedata<-list(Solvent=c(0.1331,0.1323,0.1275,0.1275,0.1184,0.1237,0.116,0.0995,0.0995,0.0968,0.0997,0.
0921,0.1016,0.0973,0.1008,0.1028,0.1937,0.1931,0.1945,0.1924,0.1936,0.1922),
Biorep=c(1,1,1,1,1,1,1,2,2,2,2,2,2,2,2,3,3,3,3,3,3),
NC=c(1.0665,0.9889,1.0056,0.9939,0.9718,0.9285,1.0537,1.0043,1.0278,1.1171,1.1137,1.1465,1.1589,1.121
2,1.0924,1.2295,1.1762,1.2322,0.7171,0.7085,0.7259,0.6816,0.6819,0.6831,0.7026,0.7024,0.6986),
PlateNC=c(1,1,1,1,1,1,1,1,1,2,2,2,2,2,2,2,2,2,3,3,3,3,3,3,3,3,3),InsertNC=c(1,1,1,2,2,2,3,3,3,1,1,1,2,2,2,3,3,3
,1,1,1,2,2,2,3,3,3),
measure=c(1.0684,1.0537,0.9642,1.0677,0.9511,0.9423,1.0096,1.0502,1.0162,0.9863,0.966,0.929,1.1151,1.
0027,1.0638,1.0827,1.155,1.1005,0.7306,0.7318,0.736,0.7489,0.7512,0.7497,0.6063,0.6063,0.6021,1.0684,1.
0537,0.9642,1.0677,0.9511,0.9423,1.0096,1.0502,1.0162,0.9863,0.966,0.929,1.1151,1.0027,1.0638,1.0827,1.
155,1.1005,0.7306,0.7318,0.736,0.7489,0.7512,0.7497,0.6063,0.6063,0.6021),
parameter=c(1,1,1,1,1,1,1,1,1,1,1,1,1,1,1,1,1,1,1,1,1,1,1,1,1,1,1,2,2,2,2,2,2,2,2,2,2,2,2,2,2,2,2,2,2,2,2,2,2,2,
2,2,2),
Plate=c(1,1,1,1,1,1,1,1,1,2,2,2,2,2,2,2,2,2,3,3,3,3,3,3,3,3,3,1,1,1,1,1,1,1,1,1,2,2,2,2,2,2,2,2,2,3,3,3,3,3,3,3,3,3)
,
Insert=c(1,1,1,2,2,2,3,3,3,1,1,1,2,2,2,3,3,3,1,1,1,2,2,2,3,3,3,1,1,1,2,2,2,3,3,3,1,1,1,2,2,2,3,3,3,1,1,1,2,2,2,3,3,3
),NP=3,NI=3)
###############
##### OpenBUGS code and inits

lineinits<-function(){list(slvmu=c(0,0,0))}

linemodel <- function() {xinsig~dnorm(0,0.0016)%_%I(0.001,)
                    chsqnsig~dgamma(0.5,0.5)
                   sigNC<-xinsig/sqrt(chsqnsig)
```

```
for(i in 1:NP){slvmu[i]~dnorm(0,1.0E-5)
                    xins[i]~dnorm(0,0.0016)%_%I(0.001,)
                    chsqns[i]~dgamma(0.5,0.5)
                    slvtau[i]<-xins[i]/sqrt(chsqns[i])}
for(i in 1:22){Solvent[i]~dnorm(slvmu[Biorep[i]],slvtau[Biorep[i]])}

for(i in 1:NP){xinsNC[i]~dnorm(0,0.0016)%_%I(0.001,)
                    chsqnsNC[i]~dgamma(0.5,0.5)
                   tauNC[i]<-xinsNC[i]/sqrt(chsqnsNC[i])
                   NCmu[i]~dnorm(0,1.0E-5)}
for(i in 1:NP){
for(j in 1:NI){NCmean[i,j]~dnorm(NCmu[i],sigNC)}}
for(i in 1:27){
NC[i]~dnorm(NCmean[PlateNC[i],InsertNC[i]],tauNC[PlateNC[i]])}

for(i in 1:1){xinsigm[i]~dnorm(0,0.0016)%_%I(0.001,)
                    chsqnsigm[i]~dgamma(0.5,0.5)
                   sigm[i]<-xinsigm[i]/sqrt(chsqnsigm[i])
for(j in 1:NP){xinsmeas[i,j]~dnorm(0,0.0016)%_%I(0.001,)
                    chsqnsmeas[i,j]~dgamma(0.5,0.5)
                   taumeas[i,j]<-xinsmeas[i,j]/sqrt(chsqnsmeas[i,j])
                   measmu[i,j]~dnorm(0,1.0E-5)}}
for(i in 1:1){
for(j in 1:NP){
for(k in 1:NI){measmean[i,j,k]~dnorm(measmu[i,j],sigm[i])}}}

for(i                                                                                                    in
1:27){measure[i]~dnorm(measmean[parameter[i],Plate[i],Insert[i]],taumeas[parameter[i],Plate[i]])}
for(i in 1:1){
for(j in 1:NP){
                    viabmu[i,j]<-(measmu[i,j]-slvmu[j])/(NCmu[j]-slvmu[j])*100}}
#for(i in 1:NP){viabdif[i]<-viabmu[1,i]-viabmu[2,i]}

##############################################

#Tf~dcat(Pf[])
#Pf[1:NP]~ddirich(alphaf[])
#for(i in 1:NP){alphaf[i]<-1}
#viabdiffin<-viabdif[Tf]

T~dcat(P[])
P[1:NP]~ddirich(alpha[])
```

```
for(i in 1:NP){alpha[i]<-1}
for(i in 1:1){viabmufin[i]<-viabmu[i,T]}

}

  ###### run the OpenBUGS
lineout<-bugs(data=linedata,inits=lineinits,digits=5,parameters=c("viabmu","viabmufin","measmean
","measmu"),model.file=linemodel,n.chains=1,n.iter=40000,n.burnin=25000,n.thin=10,debug=T)
##### print output
attach.bugs(lineout) ## imports the statistics
## in the output: viabmu are the % viability values for the two parameters, viabdif is the difference,
## viabmufin and viabdiffin are the Linear Pool consensus values for these parameters.
#####
print (lineout, digits=3)
```

Supplemental Results

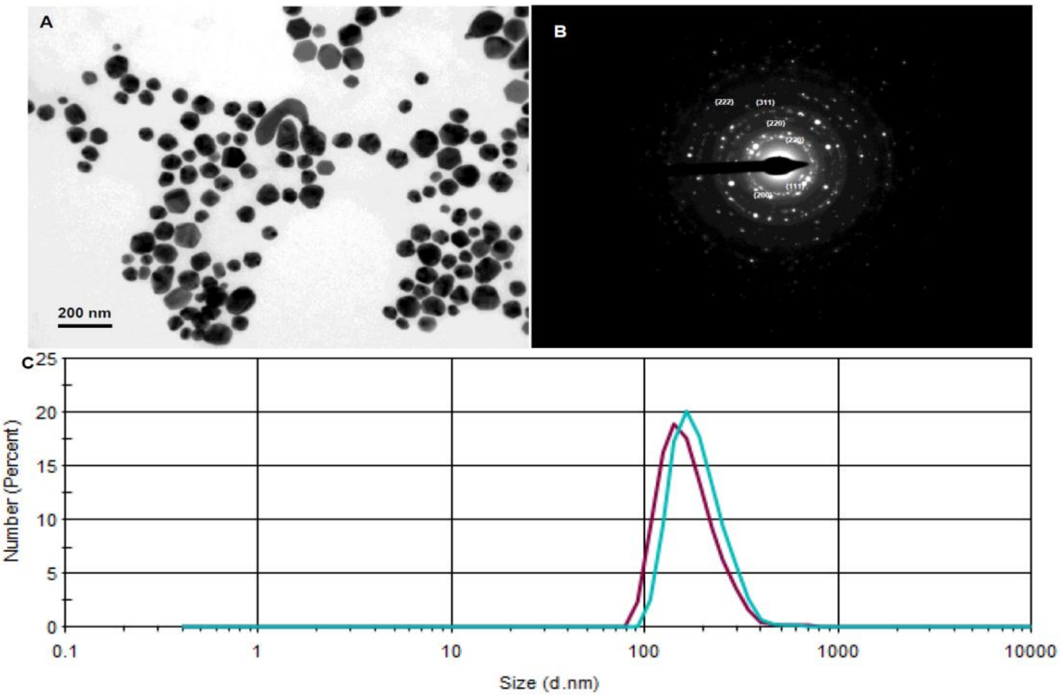

Figure S1. Nanoparticle size characterization. (A) TEM image shows the size and shape of CeO_2 NPs. (B) Selected area electron diffraction (SAED) of CeO_2 NPs. (C) Dynamic light scattering (DLS) analysis of the particle diameter calculated via size distribution by numbers. (n = 3 = three independent experiments, each experiment was performed in triplicates). Out of three, two of them were overlapping and the 3rd one covered the purple one.

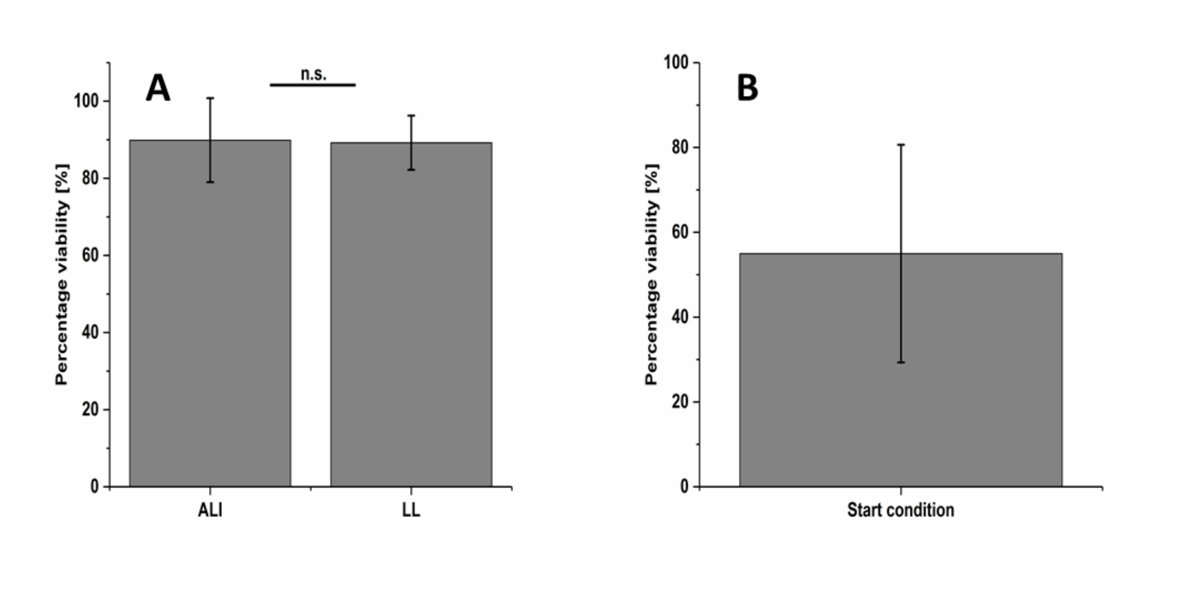

Figure S2. A549 cell viability. (A) shows cell viability under ALI and submerged (LL) culture conditions (no ALI exposure) compared to ALI cultured cells. (B) exhibits the cell viability after 1 h ALI clean air exposure compared to the incubator control without any optimization of the ALI system (initial conditions). Percentage viability values are the consensus values calculated for each of the three plates using the Bayesian modeling. The values are the means and the error bars the standard deviations.

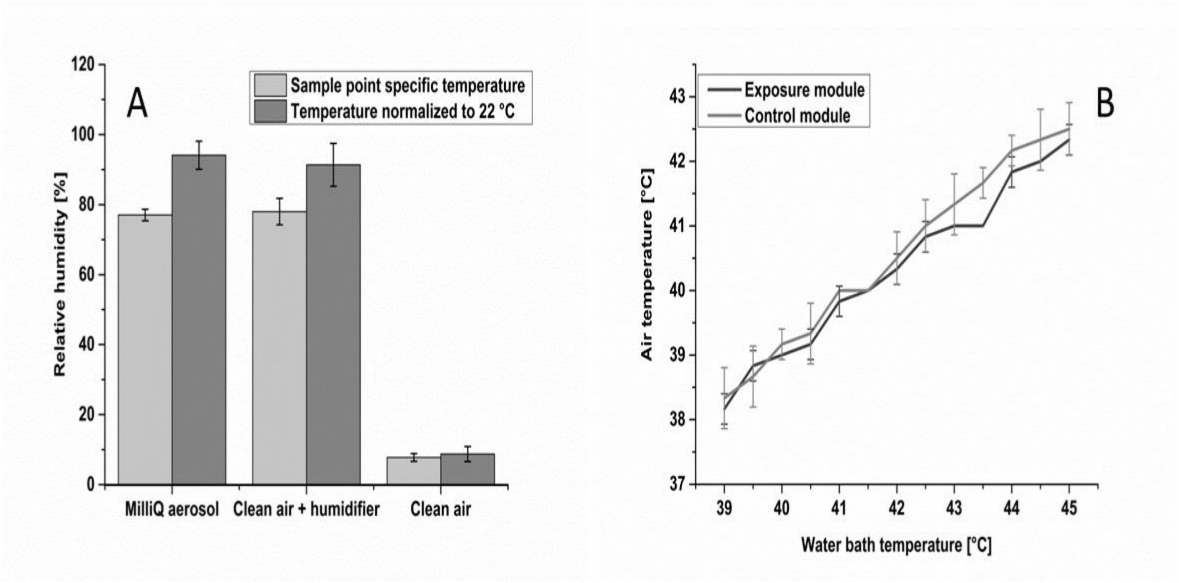

Figure S3. Characterization of the Vitrocell exposure 12/3 CF module. The relative humidity of the exposure air with and without humidification of the clean air, or a water droplet aerosol (MilliQ aerosol), which was used for particle exposure, is shown in (A). Heating the lid temperature to a certain degree does not necessarily mean that the aerosol flow temperature is equal to the lid temperature. Therefore, the relationship between the temperature of the air flow and the water bath temperature, which was used to heat the lid, is shown in part (B) for the particle exposure module and the control module (clean air only). n = 3 independent experiments with 1 technical replicate each. The values are the means and the error bars the standard deviations.

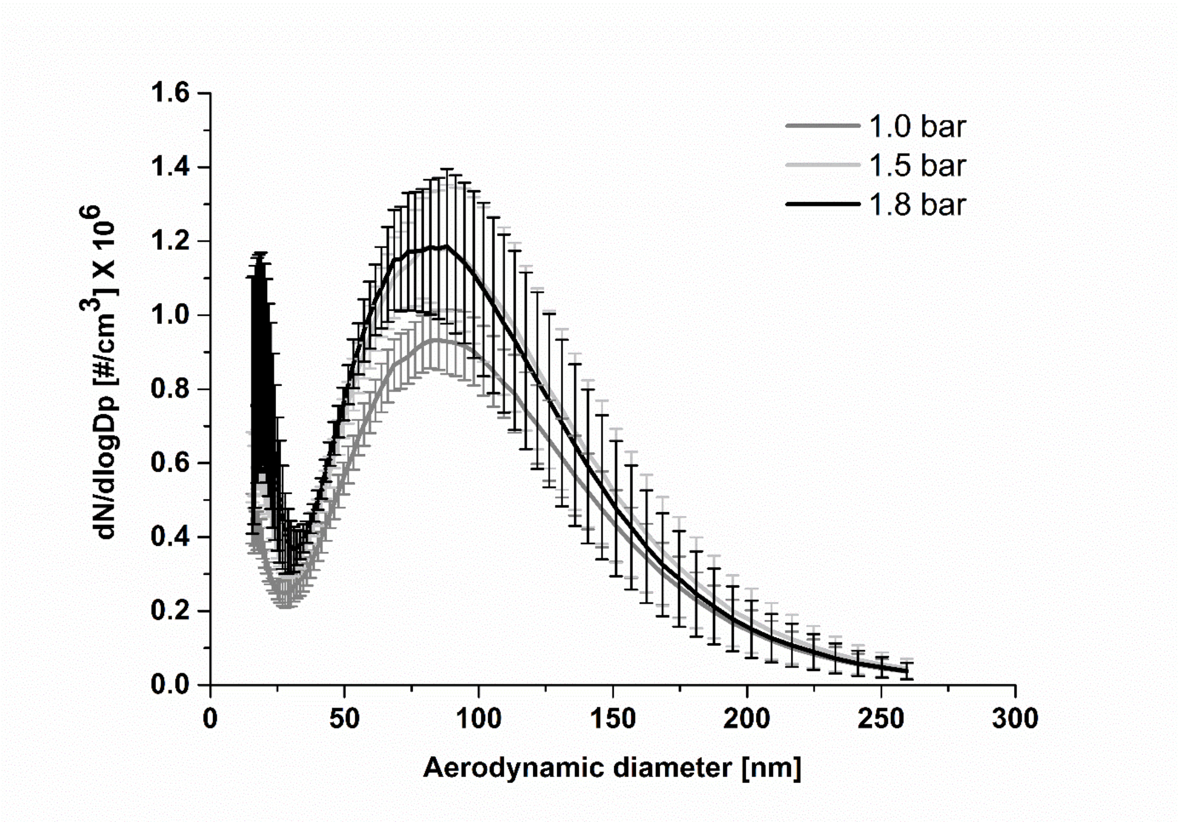

Figure S4. The particle size distribution of NM-212 as a function of the inlet pressure of the aerosol generator (n = 9 = three independent experiments with 3 data points each. Data from each experiment are combined and shown as mean ± SD).

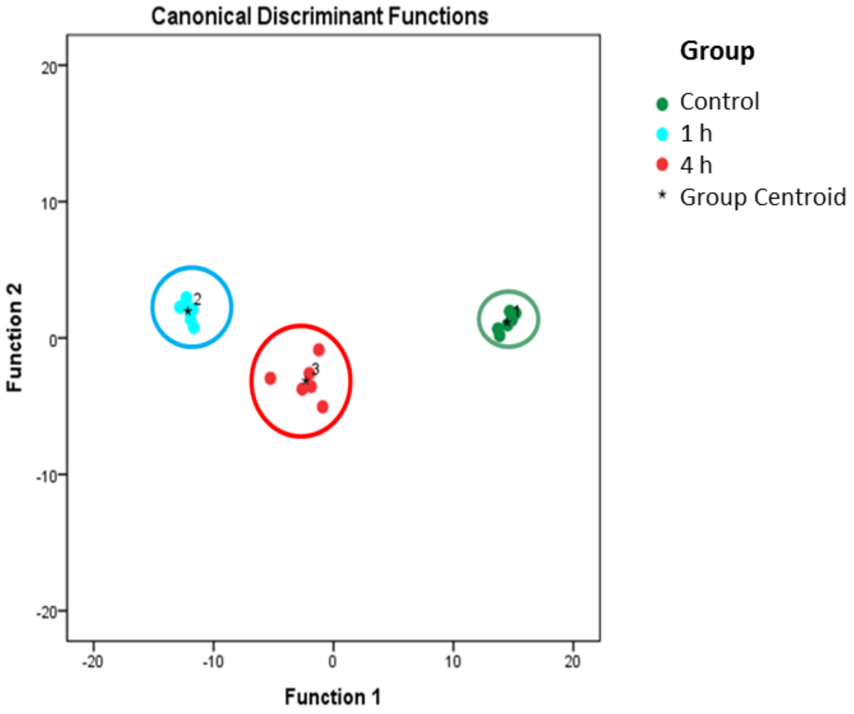

Figure S5. Fisher's linear discriminant of ToF-SIMS analysis (n = 6). Different groups show distinct differences.

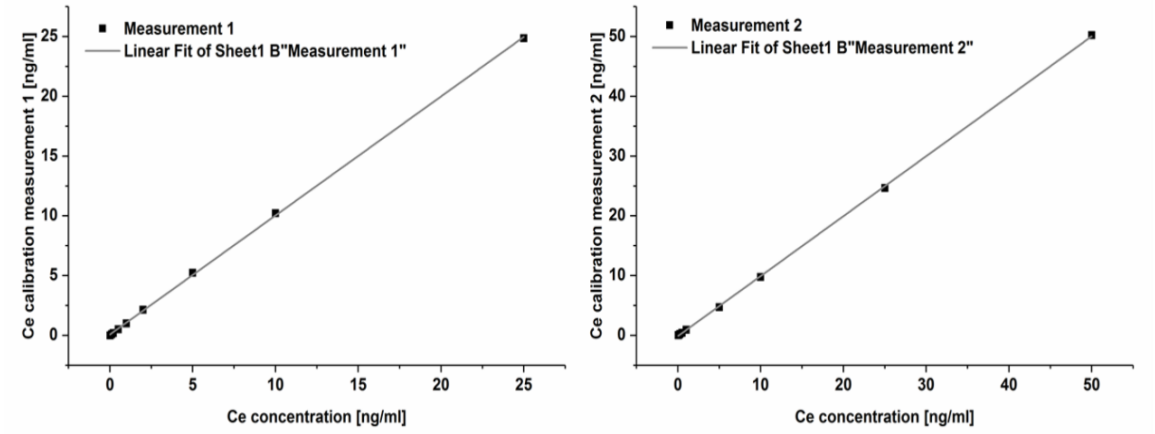

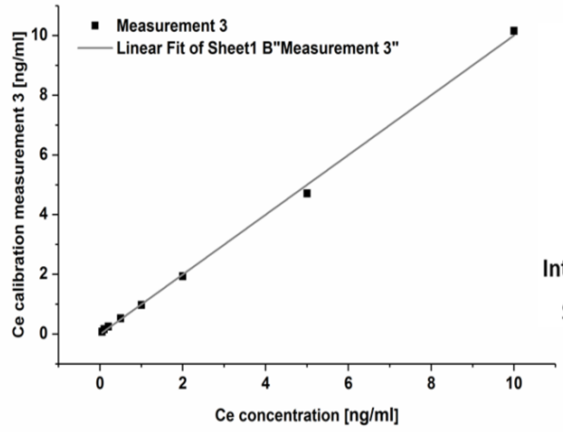

	Measurement 1	Measurement 2	Measurement 3
Pearsons´r	0.99989	0.9999	0.99931
Adj. R-Squar	0.99975	0.9999	0.99838
Intercept Value	0.07699	-0.1101	-0.00713
Slope Value	0.99553	1.0031	1.00104
Intercept Standard Error	0.05068	0.06687	0.06145
Slope Standard Error	0.00553	0.00352	0.01523

Figure S6. ICP-MS calibration curves of the three different measurements. The solid lines indicate a linear regression fit to the data.

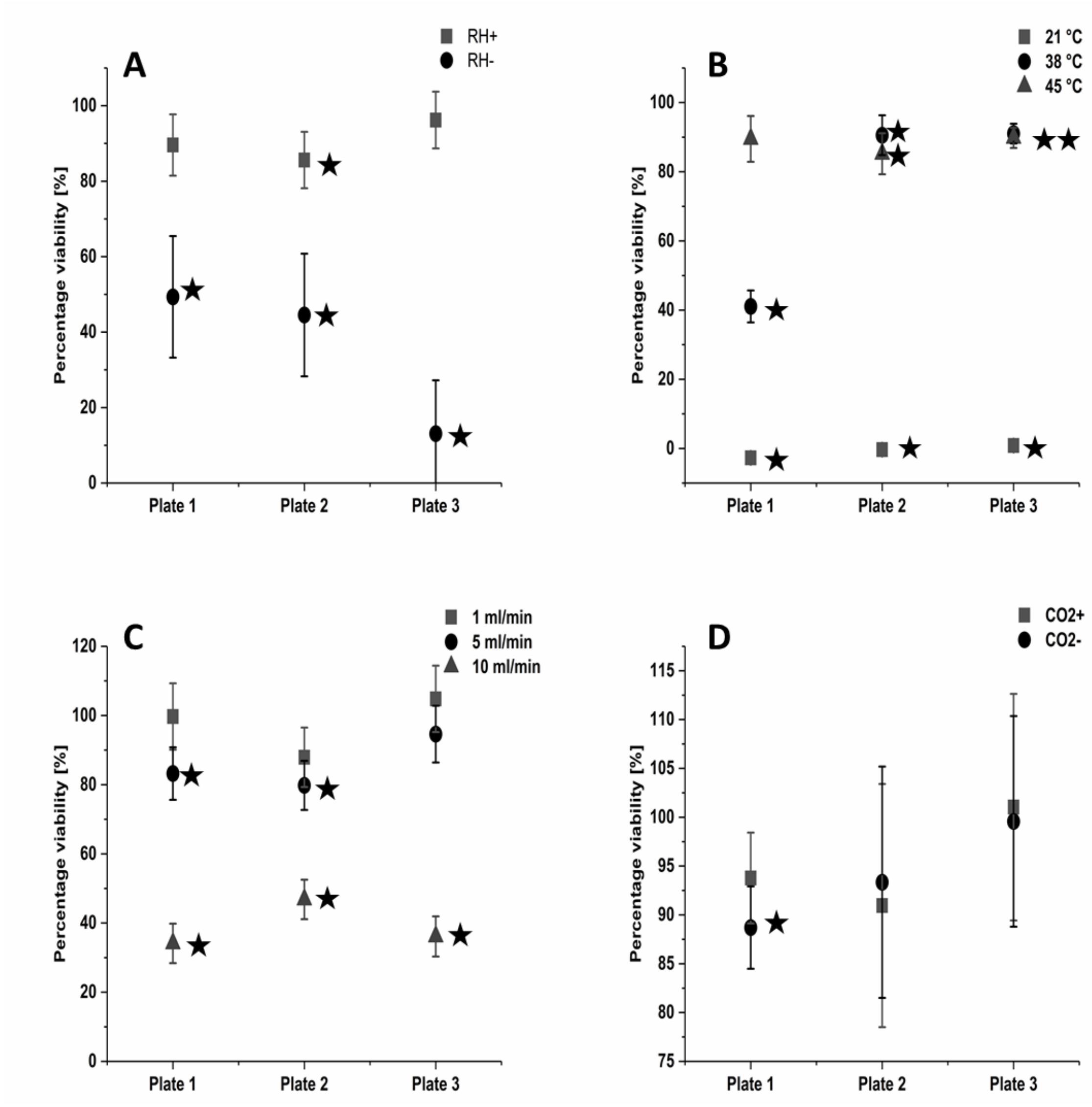

Figure S7. Plate to plate analysis of consensus values for the different parameters affecting cell viability: Relative humidity (A), lid temperature (B), flow rates (C) and 5 % CO_2 supply (D). Percentage viability values are the consensus values calculated for each of the three plates using the Bayesian modeling. The values are the means and the error bars the standard deviation. Black asterisks indicate that the consensus value is significantly less than the incubator control with a 95 % likelihood using the Bayesian modeling.

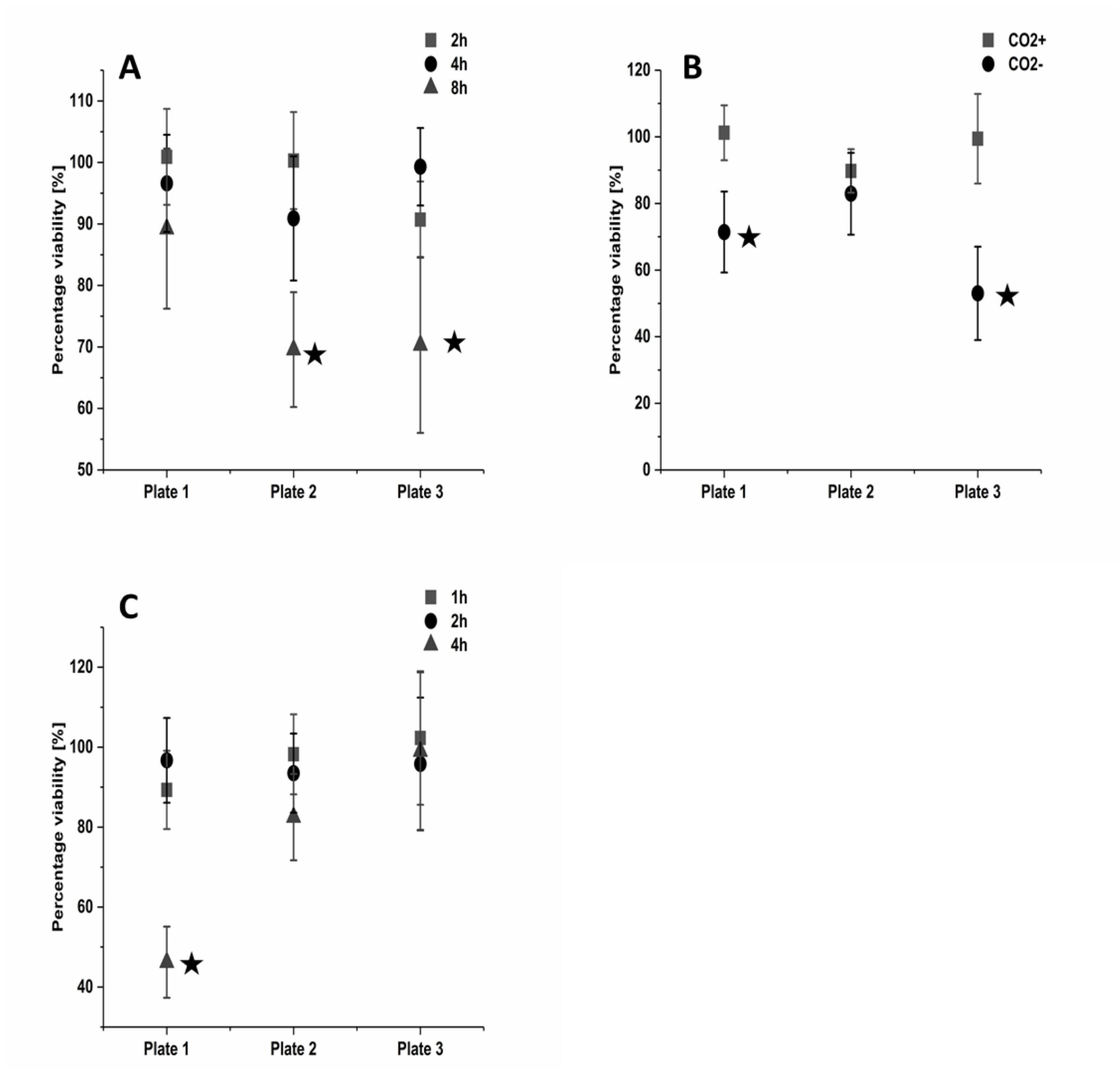

Figure S8. Plate to plate analysis of consensus values for the different parameters affecting cell viability: (A) Time-dependent cell viability for MilliQ water aerosol exposure (without ENM) using the optimized Vitrocell setup. (B) Effect of 5 % CO_2 supply to the air on the cell viability after 4 h MilliQ water aerosol exposure using the optimized Vitrocell exposure setup. (C) A time-dependent NM-212 exposure compared to MilliQ water aerosol (without NP) exposed cells. Percentage viability values are the consensus values calculated for each of the three plates using the Bayesian modeling. The values are the means and the error bars the standard deviation. Black asterisks indicate that the consensus value is significantly less than the incubator control with a 95 % likelihood using the Bayesian modeling.

Table S1. Mycoplasma test, PCR compounds.

**Material**	**Manufacture**	**Article number**	**Sequence**
TaqDNA polymerase	New England Biolabs	M0320L	
Forward Primer			5′-ggC GAA Tgg gTg AgT AAC ACg-3′
Reverse Primer			5′-Cgg ATA ACg CTT gCg ACC TAT g-3′
dNTP set (nuleotides)	Fermentas	R0182	
Genomic DNA extract from mycoplasma fermentans (positive control)	Minerva	51-0117	
GeneRuler™ 1 kb Plus DNA Ladder	Fermentas	SM1331	

Table S2. Mycoplasma test, PCR conditions and protocol.

**Temperature [°C]**	**Time [seconds]**	**Comment**
94	300	Initial denaturation
55	105	Initial denaturation
72	45	Initial denaturation
3 cycles of the following three steps		
94	45	
55	105	
72	180	
40 cycles of the following two steps		
94	45	
55	45	
1 cycles of the following three steps		
72	10	
27	10	
4	infinite	

Table S3. ICP-MS LOD/LOQ levels of the three different measurements.

Measurement	LOD [ng/ml]	LOQ [ng/ml]	Background levels [ng/ml]	R-values
1	1.22 x 10^{-4}	2.75 x 10^{-4}	5.10 x 10^{-5}	0.99969
2	6.29 x 10^{-3}	1.73 x 10^{-2}	1.57 x 10^{-3}	0.99969
3	7.19 x 10^{-2}	7.30 x 10^{-2}	7.15 x 10^{-2}	0.99931

Table S4. Possible pitfalls during an ALI exposure experiment.

Pitfalls	Troubleshooting/Advise
Chamber incompletely/not fully closed.	Connect all gas exit tubes together and check flow rates. The flow rate entering the system should be equal to the sum of the three insert flow rates.<br>Check the seal ring for any damage as well as its correct position.<br>Check/correct tightness of seal clamps.
Retention of cleaning solution (ethanol, H_2O) inside the chamber.	Wash two times with 5 ml medium before adding medium for the exposure to remove ethanol completely.
Too much medium inside the chamber or inhomogeneous membrane covering of the medium at basolateral side.	If medium is rising above the insert at the edges, remove excessive medium, and then check whether the chamber is leveled.
Not enough medium inside the chamber.	Check whether membrane is in contact with the medium, and then add medium if needed.
Incomplete cell monolayer.	If microscopic analyses before transferring into ALI exposure system reveals that the monolayer is not complete; consider adjusting the ALI culture protocol (extending growth time, increasing number of cells).
Low cell viability because of incorrect flow rate or no flow into the exposure chamber.	Flow rate should be checked before placing cells inside the chamber (valve positions of the vacuum pump can change day to day between experiments due to strong vibration of the vacuum pump).<br>The connection has to be proven after checking for any leakages every time before starting the exposure.
Low cell viability because of no humidifier connection.	The connection should be proven after checking for any leakages every time before starting the exposure.
Gas supply runs out during exposure.	If possible, switch from gas bottles to house gas lines to avoid disruption of gases due to empty bottles during exposure.<br>Filter house gas by a hydrocarbon filter and a molecular sieve to remove generator oil.

	Alternatively, replace the gas bottle earlier to avoid running out during a cellular exposure.
The gas has an overly high pressure in the chamber.	Reduce generator pressure/aerosol flow to avoid mechanical cell damage by the air flow.
Cell monolayer is not present in the center of the monolayer after exposure.	One potential cause is that the air inlet is too close to the cells. In this case, adjust inlet height until no cell damage is visually observed (i.e., cells may not be present in the middle of the insert).<br>Another potential cause is that the insert flow rate is too high. In this case, reduce the flow rate until no cell damage is visually observable.<br>A third potential cause is insufficient humidity. In this case, check the relative humidity.
Contamination of cells occurs during the exposure experiment.	Routinely clean the chamber before and after use.<br>Add antibiotics and fungicides to the cell culture medium.<br>Perform the whole experiment under sterile conditions if possible.
SMPS particle contamination observed.	Before measuring a particle size distribution, a control clearance check by performing a standard measurement with a HEPES filter in front of the SMPS air inlet should be performed.<br>If particles are detected, then clean device to remove old particles by setting the sheath flow to 30 ml/min using the HEPA filter in front of the SMPS air inlet. Then, conduct a control clearance check again. No particles should be detected after the system is cleaned.<br>Clean device after use by setting the sheath flow to 30 ml/min for a minimum of 30 minutes, using the HEPA filter in front of the SMPS air inlet.
No deposition detectable.	Air inlet possibly too far away from the cells. Try performing deposition measurements on an empty insert and quantifying the deposition.
